# Pilot tone-based prospective correction of respiratory motion for free-breathing myocardial T1 mapping

**DOI:** 10.1007/s10334-022-01032-4

**Published:** 2022-08-03

**Authors:** Juliane Ludwig, Kirsten Miriam Kerkering, Peter Speier, Tobias Schaeffter, Christoph Kolbitsch

**Affiliations:** 1 Physikalisch-Technische Bundesanstalt (PTB), Braunschweig and Berlin, Abbestr. 2-12, 10587 Berlin, Germany; 2 Siemens Healthcare, Erlangen, Germany; 3 Department of Biomedical Engineering, Technische Universität Berlin, Berlin, Germany

**Keywords:** Prospective respiratory motion correction, T1 mapping, Pilot tone, Cardiovascular MR

## Abstract

**Objective:**

To provide respiratory motion correction for free-breathing myocardial T1 mapping using a pilot tone (PT) and a continuous golden-angle radial acquisition.

**Materials and methods:**

During a 45 s prescan the PT is acquired together with a dynamic sagittal image covering multiple respiratory cycles. From these images, the respiratory heart motion in head-feet and anterior–posterior direction is estimated and two linear models are derived between the PT and heart motion. In the following scan through-plane motion is corrected prospectively with slice tracking based on the PT. In-plane motion is corrected for retrospectively. Our method was evaluated on a motion phantom and 11 healthy subjects.

**Results:**

Non-motion corrected measurements using a moving phantom showed T1 errors of 14 ± 4% (*p* < 0.05) compared to a reference measurement. The proposed motion correction approach reduced this error to 3 ± 4% (*p* < 0.05). In vivo the respiratory motion led to an overestimation of T1 values by 26 ± 31% compared to breathhold T1 maps, which was successfully corrected to an average difference of 3 ± 2% (*p* < 0.05) between our free-breathing approach and breathhold data.

**Discussion:**

Our proposed PT-based motion correction approach allows for T1 mapping during free-breathing with the same accuracy as a corresponding breathhold T1 mapping scan.

**Supplementary Information:**

The online version contains supplementary material available at 10.1007/s10334-022-01032-4.

## Introduction

Cardiovascular magnetic resonance imaging (CMR) helps physicians to diagnose a variety of pathological changes in the myocardium [1–3]. Commonly, different MR imaging techniques are applied in separate scans during a clinical exam [4]. Measurements of the spatial distribution of the T1 relaxation time (T1 mapping) allow assessment of ischemic and nonischemic cardiomyopathies [5] like fibrosis [6, 7], amyloidosis [8], and iron overload [9]. Cine imaging is used to capture cardiac wall motion abnormalities and quantitatively assess cardiac function [10].

Common techniques for myocardial T1 mapping include MOLLI and SASHA [11, 12]. For these techniques, after an inversion or saturation pulse one image per cardiac cycle is acquired at different inversion or saturation times. A model is then fitted to these images to calculate the T1 times for each voxel. Usually, T1 maps are recorded under breathhold conditions and prospective ECG triggering. But acquiring an ECG signal can be difficult, due to magnetohydrodynamic effects or complex QRS patterns of the patient, and mistriggering leads to artifacts [13, 14]. To avoid these problems a continuous acquisition can be used, where data is optimized or resorted retrospectively, as proposed for T1 mapping with simultaneous cine imaging by Zhou et al. [15].

However, for cardiac imaging respiratory motion is a major cause of artifacts. Although patients are asked to hold their breath, previous studies on T1 mapping have shown that respiratory motion of the heart was still present in 40% of patients due to limited breathholding capability [16].

Many retrospective motion correction strategies have been proposed that allow free-breathing acquisitions and compensate for respiratory motion by realigning the obtained qualitative images before T1 map reconstruction [17–20]. A common feature of these approaches is that they can only correct for in-plane motion and not through-plane motion. To achieve through-plane motion correction for 2D imaging, slice tracking approaches are required, which update the slice position in real-time during data acquisition. Until now, slice tracking for free-breathing T1 mapping is based on using an MR navigator [21–23]. However, if a continuous sequence is required, e.g. to reconstruct multiple cardiac phases retrospectively, the use of an MR navigator is not suitable. Regardless of the acquisition time of the image-based navigator, it would interrupt the continuous measurement.

An alternative to the MR-navigator is the use of the pilot tone (PT) as a motion surrogate. The PT is currently an active field of research with constant advancements [22–27]. So far it has been used for a wide range of different applications as a scale-free motion surrogate but very little as a quantitative surrogate [28–30]. The PT is very versatile, and an advantage lies in the applicability for continuous scans, i.e., it can be acquired without interrupting the data acquisition. Recently it has been shown that the PT can be used for prospective motion correction for Cartesian 2D cine imaging [31].

Here we propose the use of the PT for a prospective respiratory motion correction during myocardial T1 mapping with a continuous radial acquisition scheme [32]. The PT is calibrated to the respiratory motion via a prescan. After calibration, the PT provides a quantitative motion surrogate that enables adaptation of the slice position to the respiratory motion of the heart in real-time [31]. The PT-based motion correction approach is extended by a retrospective non-rigid motion correction.

Our new technique is used for high-resolution free-breathing myocardial T1 mapping using the radial sequence proposed in [32]. With this sequence, T1 maps of different cardiac phases can be reconstructed. In this paper, the focus is on mid-systole and mid-diastole. A feasibility study of the method was conducted in a motion phantom and 11 healthy subjects.

## Materials and methods

**Fig. 1:**
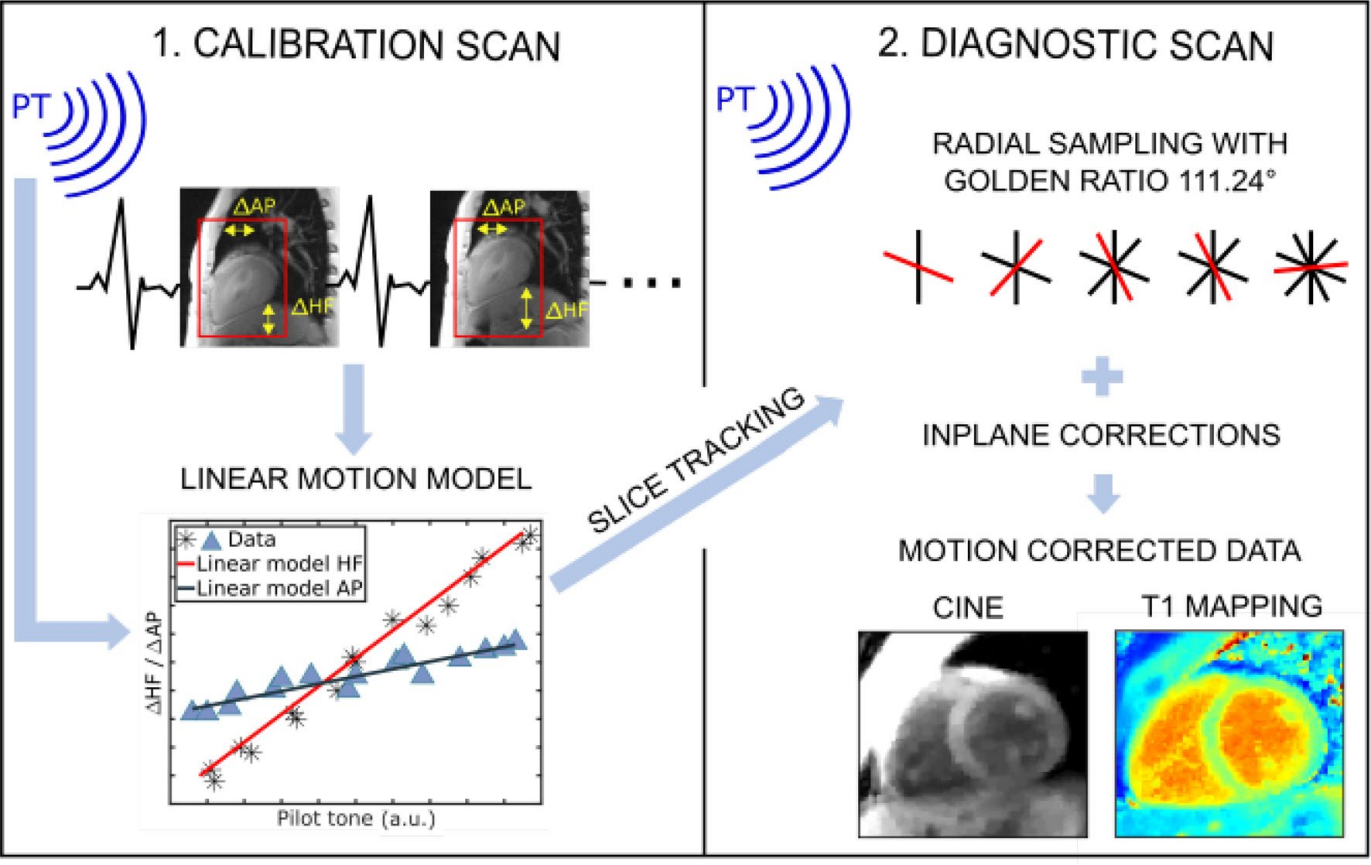
The motion correction method with the PT consists of two steps, the calibration scan, and the T1 mapping scan. During the calibration, HF and AP motion of the ROI covering the heart are registered and correlated with the PT by two motion models. During the motion corrected radial T1 mapping scans, the RF excitation pulse is adjusted for every readout to follow the heart motion during the scan (slice tracking). In-plane motion correction is applied to the acquired k-space data retrospectively.

An overview of our method is given in Fig. 1. For the proposed motion correction approach, the PT is first calibrated to the motion of the heart via a calibration scan that consists of sagittal ECG-triggered images, one image acquired per cardiac cycle over several breathing cycles. Two motion models for HF and AP direction are derived from the calibration. With the calibrated PT signal the respiratory motion in HF and AP direction is estimated for every readout during the subsequent scans. Through-plane motion correction is applied during measurements by changing the frequency of the RF pulse and thus enabling prospective slice tracking. The resulting translational in-plane shifts derived from the PT signal and the motion models are applied retrospectively before reconstruction to the k-space data. Additionally, non-rigid image registration is used retrospectively to estimate and correct any residual motion that might not be captured by the PT.

### Pilot tone

The PT is a coherent, continuous RF signal produced by a commercial RF-synthesizer (ESG 1000A; Hewlett Packard, Palo Alto, CA) connected to an inhouse-built non-resonant antenna and transmitted into the bore of the MR scanner [25]. The used power for the PT generation is in the order of magnitude of the spin signal, and 8 orders of magnitude smaller than the average power of MR transmit pulses, which are limited according to the standard MR safety guidelines to 2 W/kg body weight [33]. Because the coil load of the receiver coils changes due to underlying physiological motion [34–37], the received intensity of the PT signal varies. The PT is therefore suitable for respiratory motion detection and has previously been compared to other motion surrogates, i.e., respiratory sensors or MR-navigators [29, 31, 38, 39].

The frequency of the PT is set, such that the signal can be recorded in the two-fold oversampled region of the field of view (FOV). The PT is obtained simultaneously with each readout without interfering with the MR signal of the object. When the RO lines are sorted by time of recording, a 1D Fourier transform of k-space along the RO direction results in the PT appearing as a continuous line with varying intensity over time in the oversampling region. Similar to the approach presented by Speier et al. [24], a reference signal is created and then fitted to a model $A*\mathrm{exp}(i2\pi ft)$ by multiplying the complex conjugate of the model with the k-space data. The complex amplitude A is logged as the PT. The fitted reference signal is subtracted from the measured k-space data, which reduces the intensity of the PT in the data before image reconstruction. To ensure greater temporal stability of the PT signal, a median filter with a sliding window step size of 100 data points was applied to the PT introducing a maximal delay of 0.6 s [31].

### Continuous radial acquisition

**Fig. 2:**
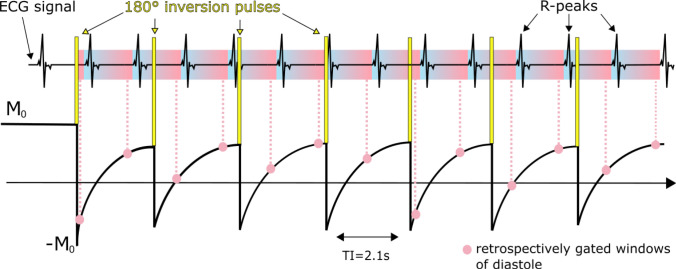
Magnetization curve for continuously acquired data with seven inversion RF-pulses. In this scheme, the pink dots represent retrospectively gated data, indicating that only data from mid-diastole are used for T1 reconstruction. Because the acquisition is not gated T1 maps for any cardiac phase (ranging from the blue to the pink area) can be reconstructed

To obtain T1 maps, a continuous non-gated golden-angle radial acquisition is used [32]. Seven inversion pulses, indicated as yellow bars in Fig. 2 are applied repeatedly with a fixed predefined delay of 2.1 s, which has been shown to allow for accurate T1 mapping for a wide range of different heart rates [13, 16]. The start of the scan is triggered to mid-diastole, but the following inversion pulses and acquisitions are carried out independent of the cardiac phase. With this acquisition scheme T1 maps for any cardiac phase could be generated. In this paper we focus on diastole and systole for T1 mapping. From the same raw data also cine images are reconstructed that allow for differentiation between cardiac phases.

### Motion correction

For 2D imaging, in-plane motion can be corrected retrospectively, as all data are available, in comparison to through-plane motion, where data cannot be recovered.

During a ~ 45 s calibration scan the PT signal is acquired simultaneously with 2D sagittal images of the heart. Prior to the scan, a region of interest (ROI) is chosen manually by using the shimming box in the user interface. Translational heart motion in head-feet (HF) and anterior–posterior (AP) direction is estimated by applying a 2D cross-correlation function to the images that cover multiple respiratory cycles. The motion estimates and the PT are used to derive two independent linear models, that allow to quantify the PT signal. In the subsequent scans, the heart motion can be predicted from the motion models and the PT for each phase encoding point.

For the slice tracking, the slice orientation plays a major role. To be able to extract the PT at the same frequency position for all scans in the oversampling region, the prospective shift must be applied along the slice normal, which may be different from the motion direction. The adapted slice position $\Delta \overrightarrow{SL}$ is the orthogonal projection of the predicted shifts $\Delta H{F}_{pred}$ and $\Delta A{P}_{pred}$ onto the slice normal $\overrightarrow{SN}$ of the scan orientation

1 $$\Delta \overrightarrow{ SL}={P}_{\overrightarrow{SN}}\left(\overrightarrow{M}\right)$$

with $\overrightarrow{M},$ as the motion vector $$\left(\begin{array}{c}0\\ \Delta A{P}_{pred}\\ \Delta H{F}_{pred}\end{array}\right)$$.

To apply the shift, the RF pulse frequency is adjusted accordingly for each readout without processing delay during the execution of the sequence and shifts are calculated on the reconstruction computer. Translational in-plane motion is corrected retrospectively using the PT motion information by applying a phase factor

2 $${\Delta \mathrm{\varphi }}_{RO, PE}=\frac{2\uppi }{{N}_{RO, PE}}{*n}_{RO, PE}*{P}_{\overrightarrow{RO},\overrightarrow{PE}}\left(\overrightarrow{M}\right)$$

to the k-space data. ${P}_{\overrightarrow{RO},\overrightarrow{PE}}(\overrightarrow{M})$ is the projection of the motion vector $\overrightarrow{M}$ onto the readout direction vector $\overrightarrow{RO}$ and onto the phase encoding direction vector $\overrightarrow{PE}$. *N*_RO,PE_ is the total number of phase encoding points along the k-space dimension k_RO,PE_, and the index *n*_RO,PE_ describes the k-space location. Detailed information on the PT correction is given in [31]. The PT-based motion correction is optimal for regions near the ROI of the calibration scan. However, phase shifts are applied globally. The PT correction is applied for each read-out and covers through-plane and in-plane motion caused by HF and AP respiratory heart motion.

Additionally, to the rigid correction based on the PT and the motion models, a non-rigid image registration is applied to the data, which corrects residual in-plane motion, for example induced by right-left heart motion. The sorting of the k-space data into 4 motion states was performed based on the PT [29]. Non-rigid image registration using a b-spline-based algorithm with mutual information as similarity metric is carried out using regularization with a bending energy penalty [41]. The first 100 readouts were not used for the estimation of the respiratory motion fields, because the PT may not be accurate for these samples due to an applied median filter with a step size of 100 readouts [31]. This affects less than 5% of the total number of radial lines and, hence, there is still sufficient data available for motion estimation. The obtained motion fields are then applied in subsequent motion-corrected image reconstruction to further minimize respiratory motion artifacts [42]. The effect of the motion fields is determined by calculating the *R*^2^ of the T1 fit of these segments with and without applied motion fields. The motion field analysis refers to inter-image results of motion correction.

### Cine reconstruction

Cine images are reconstructed from the same raw data used for T1 mapping by resorting the radial lines into different cardiac phases based on the recorded ECG signal. The first 100 readouts acquired directly after an inversion pulse are excluded in the cine reconstruction to ensure a consistent dark blood contrast over all cardiac phases [40]. The dark blood contrast results from applying global inversion pulses in the sequence and the fact that the T1 of blood is longer than that of myocardium (1900 ms compared to 1100–1350 ms at 3 T [43, 44]). To enhance image contrast, only data are used where the signal from myocardium is already positive during relaxation. The signal of blood is not yet completely positive but still partly negative because of the longer T1, resulting in a nulling of blood signal intensities [32]. Respiratory motion fields are applied during total variation (TV) regularized respiratory motion-corrected image reconstruction [45]. This image reconstruction problem is solved iteratively, and the motion fields are included in the MR acquisition model [42]. The acquisition model, therefore, describes the weighting due to multiple receiver coils, motion transformation, Fourier encoding and radial k-space sampling. Total variation (TV) regularization is applied spatially (*λ* = 10^−3^). Coil sensitivities were calculated combining all radial data. From the reconstructed cine images the rest period in systole are visually selected.

### T1 mapping analysis

T1 mapping is carried out for a predefined cardiac phase (e.g., mid-diastole or mid-systole). One T1-weighted image per cardiac cycle is reconstructed with 43 radial lines each (corresponding to an acquisition window of 202 ms for diastole). Because the cardiac cycle and the pattern of the inversion pulses are asynchronous, the images correspond to different inversion times (TI).

T1 maps are reconstructed using the same TV-regularized image reconstruction as above. The respiratory motion fields are applied during image reconstruction to ensure all TI images are in the same respiratory phase. An extended inversion recovery Look-Locker model is used in a three-parameter fit to estimate M0, T1, and the (effective) flip angle voxel-wise [15].

Systolic cardiac phases are visually selected from the reconstructed cine images. Fluctuations in heart rate or arrhythmias may result in TI images acquired at different cardiac phases and produce inaccuracies in the T1 maps [45–47]. For such patients, the short resting phase of systole must be found very precisely and cannot always be derived from the ECG due to decreased signal quality during arrhythmia [48]. For this purpose, cine images were reconstructed, which are used as temporal scout to find the systole.

The radial sequence provides optimal results for mid-diastole, i.e., because the sequence start is in mid-diastole. To accurately determine the T1 for systole as well, the M_0_ which is included in the fitting process for the generation of the systolic T1 maps, is taken from the previously reconstructed diastolic T1 maps, as proposed by Becker et al. [12]. This approach is feasible because the M_0_ of the muscle remains the same during contraction.

### Experiments

All measurements were performed with inhouse-programmed sequences on a 3 T scanner (MAGNETOM Verio, Siemens Healthcare, Erlangen, Germany) on 11 healthy subjects (7 male, 4 female, age 30 ± 7 years old, weight 72 ± 11 kg) after approval of the local ethics board. Written informed consent was obtained in all cases. In addition, scans were carried out with an inhouse-built T1 phantom placed on a moving wagon which allowed for translational motion. The diameter of the tubes inside the phantom is 2.75 cm and the motion amplitude in HF direction is 26.7 mm. The parts of image reconstruction and evaluation, which were required for the application of the prospective correction, were implemented on the scanner (software *syngo*.MR B17). Retrospective motion correction, image reconstruction, and estimation of T1 were carried out using Python 3.7.

### Scan parameters

For calibration, 2D ECG-triggered data were acquired over 45 cardiac- and 7.2 ± 3.3 respiratory cycles in the diastolic phase in sagittal view with FOV = (320 × 320) mm^2^, voxel size = (1.7 × 1.7 × 8) mm^3^, TE/TR = 2.1/4.7 ms, and flip angle (FA) = 5° using an in‐house‐modified spoiled gradient‐echo sequence. The scan parameters (FOV, bandwidth) of the calibration scan were chosen to match the motion corrected scan so that the pilot tone is comparable between the two scans.

The T1 mapping scan was performed continuously during ~ 15 s in short axis view using an inhouse-modified 2D spoiled gradient-echo sequence with a golden-angle radial trajectory [12]. In total 3080 radial lines were acquired during 16.2 ± 2 heartbeats with FOV = (320 × 320) mm^2^, voxel size = (1.7 × 1.7 × 8) mm^3^, TE/TR = 2.1/4.7 ms and FA = 5°. Seven adiabatic inversion pulses were applied every 2.1 s independent of the cardiac cycle. The scan was carried out during free-breathing without (uncorrected) and with motion correction (corrected) and during a breathhold which served as a reference for comparison. The start of the sequence was triggered to the mid-diastole but afterwards the sequence ran freely of the cardiac cycles.

An inversion recovery spin echo sequence (TI = 25/50/300/600/1200/2400/4800 ms, TE/TR = 12/8000 ms) was used to obtain reference T1 values for the phantom. In vivo, a standard 5(3)3 MOLLI sequence with FOV = (360 × 360) mm^2^ and voxel size = (1.4 × 1.4 × 8) mm^3^ with adiabatic inversion pulses was acquired during ~ 11 s and breathhold conditions during. MOLLI data were post-processed and T1 fitted inline at the scanner [49]. Cine images with a standard gradient‐echo sequence FOV = (320 × 320) mm, voxel size = (1.7 × 1.7 × 8) mm, TE/TR = 3.22/5.9 ms, and FA = 12° were acquired.

In all subjects, a mid-ventricular short axis was obtained. Additionally, a full stack of 10 short axis images covering the left ventricle, long axis (LA) images and 4 chamber view (4CHV) images were recorded in one subject. For comparison breathhold scans are performed in the end-expiratory state. For the free-breathing and motion corrected scans, the sequences were started independently of the motion state. The motion-corrected image may, therefore, show a different position in the short axis view and could lead to differences in the quantitative comparison.

To reconstruct phantom T1 maps comparable to the in vivo measurements (i.e., reconstruct for diastole), an ECG of a subject was used for cardiac gating of the phantom data yielding 16 images.

### Analysis

For the analysis of the phantom data, nine circular ROIs were manually placed in the center of each tube with a diameter of 1.7 cm. Mean T1 values were calculated and compared between the reference scans and the uncorrected and corrected scans, respectively.

In vivo data were analyzed by segmenting the left ventricle, according to [50], and comparing T1 values of the six segments of all subjects of the breathhold data with the uncorrected and corrected data, respectively.

MATLAB R2017a and Python 3.7 were used for the analysis. A Wilcoxon test was used for statistical analysis, where p-values < 0.05 were classified as significant.

## Results

### Phantom

**Fig. 3:**
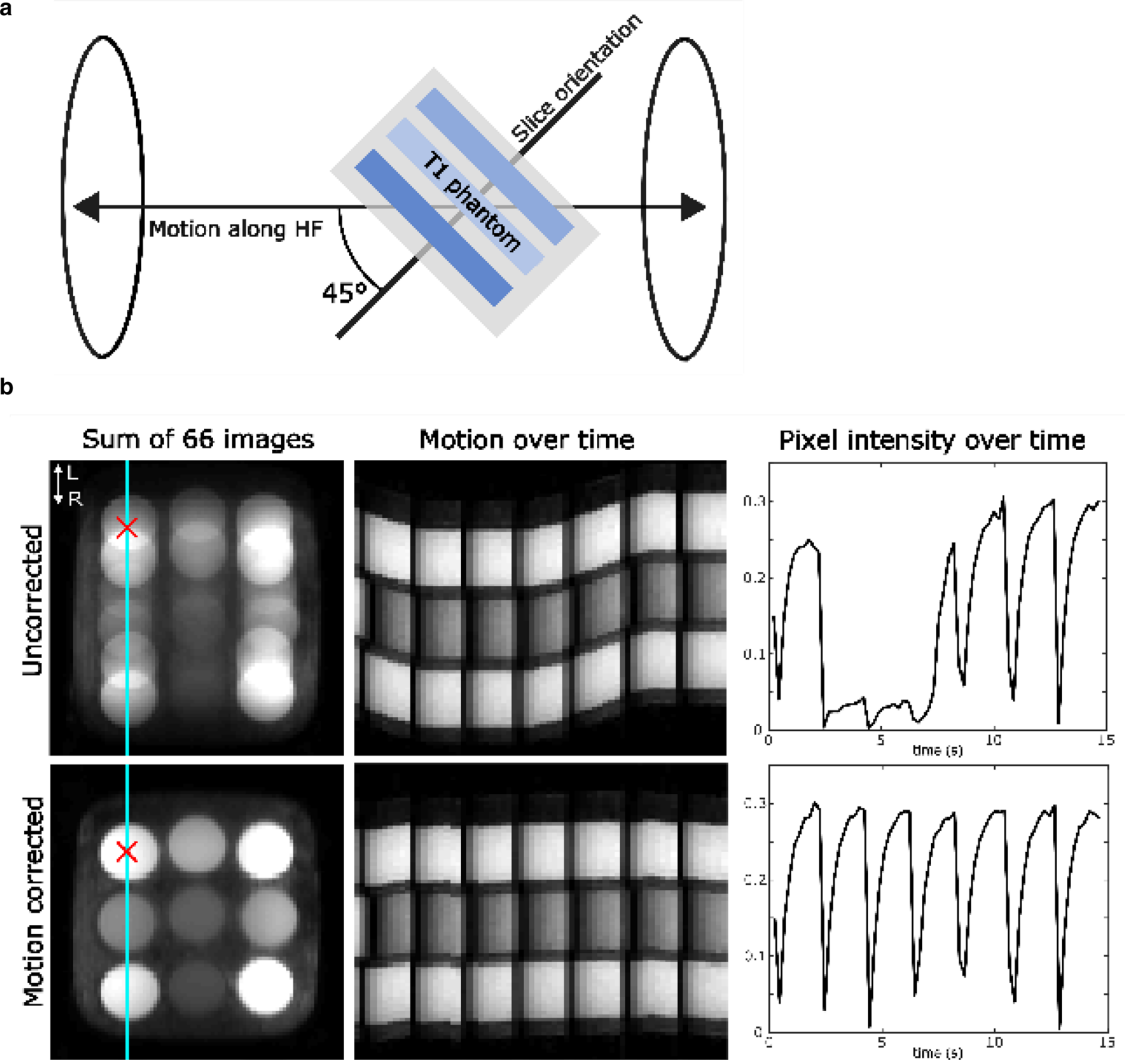
**a** Phantom setup showing the T1 phantom and slice orientation tilted to the long-axis in head-feet direction **b** Comparison of uncorrected (top) and corrected (bottom) data. Left: 66 images each reconstructed from 43 radial lines were overlayed. Middle: Single line from the left image (cyan) displayed over ~ 15 s to show the change in phantom position during measurement. Black dropouts are due to the inversion pulses. Right: Intensity change of one pixel (red cross) over ~ 15 s

**Fig. 4:**
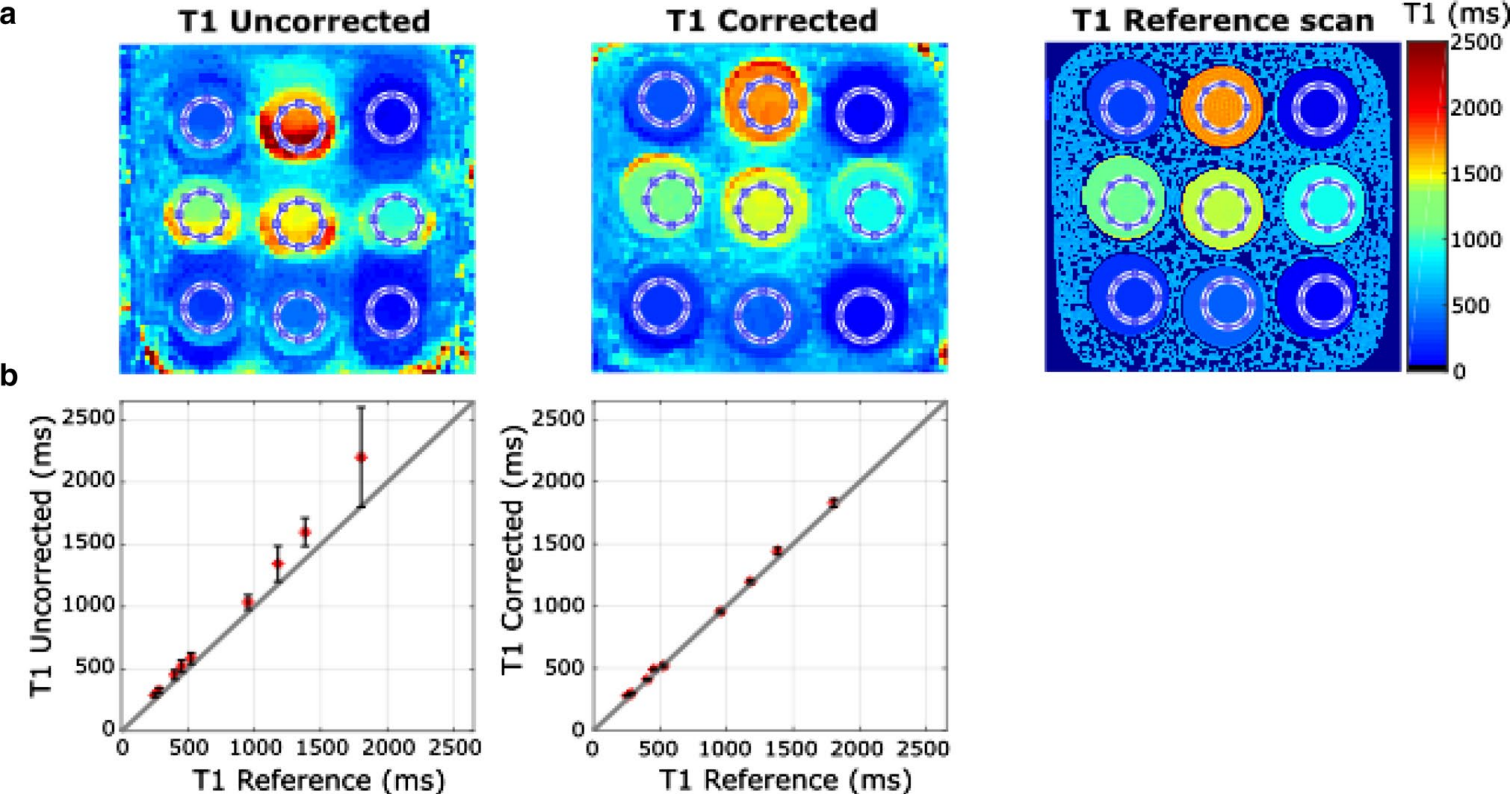
**a** T1 maps of a moving phantom without motion correction and with motion correction. For comparison, a reference scan was performed using an inversion recovery spin echo sequence. Circles indicate the ROI. **b** T1 times of the tubes for the uncorrected with 14 ± 4% difference and the corrected scans with 3 ± 4% difference compared to the reference. The gray line indicates the identity line.

Due to the angulation of the phantom, through-plane and in-plane motion were present as shown in Fig. 3a. The shift along HF estimated with the calibration scan was 21.8 mm. The correction applied during the T1 mapping scan was 15.5 mm for through-plane motion and 15.4 mm for in-plane motion in readout direction. Figure 3b gives an overview of 66 images each reconstructed from 43 radial lines with and without motion correction. The according T1 maps were estimated from 16 images. In Fig. 4 the resulting T1 maps are displayed (a) and the T1 values of the uncorrected and corrected scans were compared to the reference T1 (b). The circular ROI was manually placed in the center of each tube with a diameter of 1.7 cm, not covering the residual motion artifacts at the border of the tubes. T1 of the surrounding material is 713 ± 23 ms. T1 of the uncorrected data was significantly higher by 14 ± 4% (*p* = 0.008) than the reference T1 values. This is expected because the fit of the signal model leads to inaccurate results in the presence of motion artifacts, leading in this case to an overestimation of T1. The difference between the corrected T1 values and the reference T1 values was 3 ± 4% and was significant (*p* = 0.02).

### In vivo

T1 maps of one subject were excluded from analysis because the reference breathhold scan was mistriggered, which affected the comparability. The mean amplitude of the respiratory induced heart motion across all subjects in the two calibration directions HF and AP were 13.9 ± 6.5 and 4.8 ± 3.4 mm, respectively. The mean $\Delta \overrightarrow{SL}$ corrected during the running sequence with the PT was 7.4 ± 3.7 mm. Retrospective in-plane motion correction based on the PT was applied for a mean motion amplitude of 6.5 ± 5.2 mm. The average amplitude of the non-rigid motion fields in a ROI around the heart was 1.1 ± 0.4 mm.

**Fig. 5:**
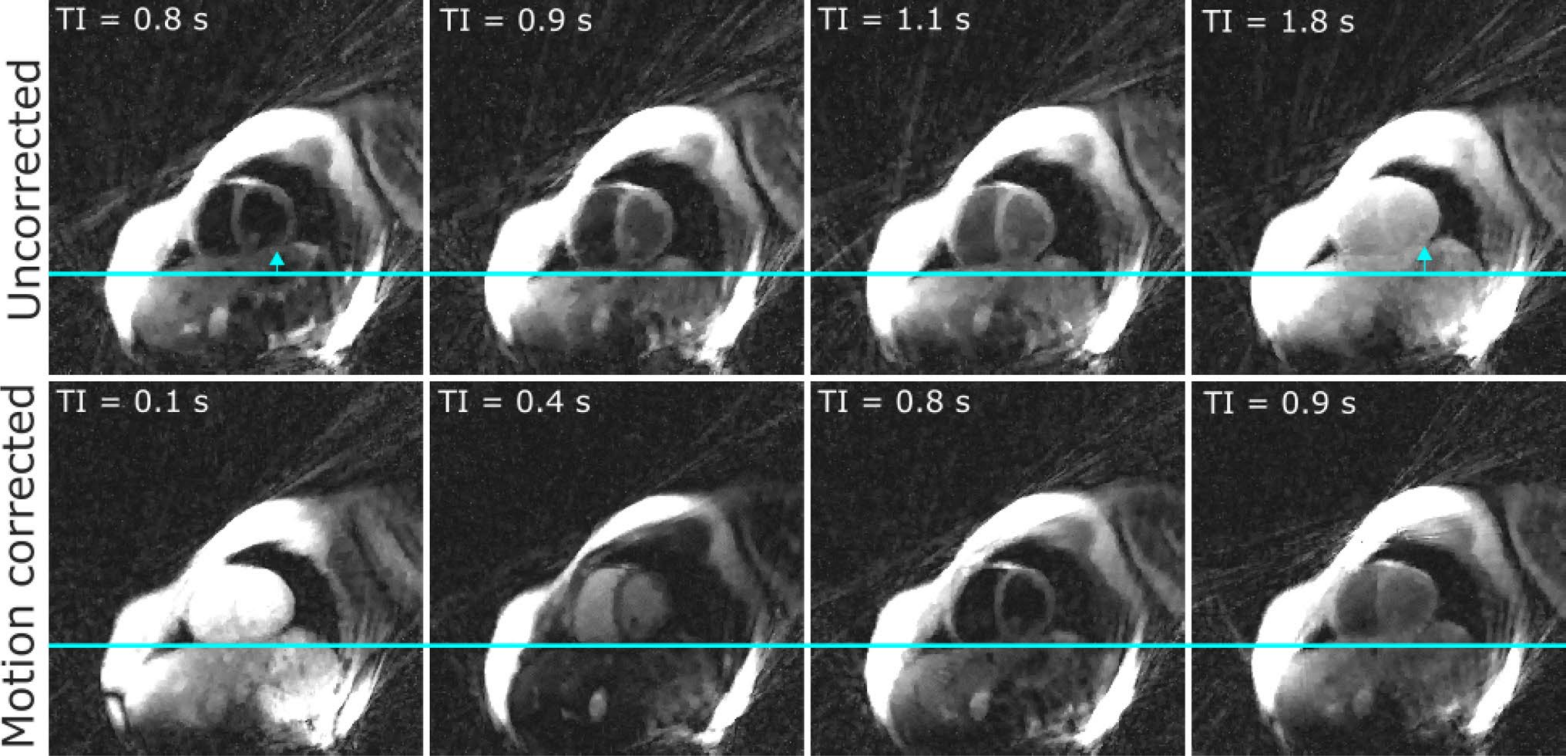
Reconstructed images from different respiratory states and different inversion times (TI in s). The motion corrected images show less heart motion as can be seen by comparison of the distance between guideline and myocardium

In Fig. 5 images of different respiratory states and inversion times were reconstructed showing the heart motion during radial acquisition of 15 s with and without motion correction.

**Fig. 6:**
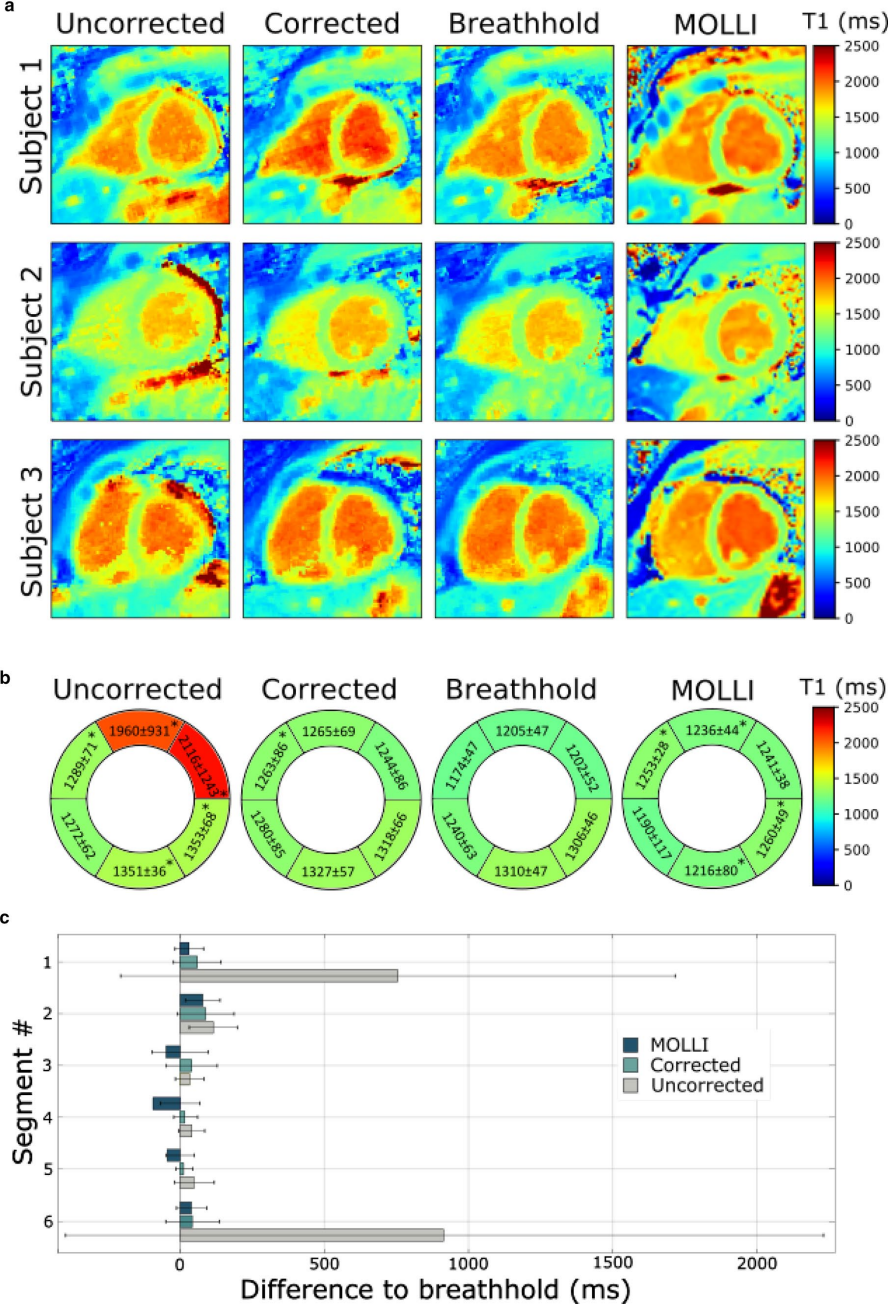
**a** Native T1 maps of three subjects acquired with a continuous radial trajectory during free-breathing without (uncorrected) and with motion correction (corrected). For comparison, T1 maps acquired during a breathhold and MOLLI (also acquired during a breathhold) are displayed. Each method was acquired in a separate scan resulting in small differences in the visualized anatomy. **b** Bull’s-eye plots representing six myocardial segments of mid-ventricular slices displaying T1 times and standard deviations averaged across 10 subjects in milliseconds. Segments marked with * are classified as significantly different. **c** Differences to reference breathhold data with errorbars for 6 segments

Figure 6a shows T1 maps of three subjects acquired during free-breathing without correction and with correction. For comparison, data were also acquired during a single breathhold at end-expiration and using MOLLI as a visual reference. 15 ± 3 T1-weighted source images go into estimating the T1 maps on average. Motion artifacts were reduced by applying our motion correction method. T1 maps are visually comparable to T1 maps obtained by the MOLLI sequence.

Figure 6b shows Bull’s-eye plots representing the mean T1 values and standard deviations of ten subjects for 6 myocardial segments. Differences of the uncorrected data to breathhold data and corrected data to breathhold data were calculated and segments marked with * are classified as significantly different. The mean values across all segments and subjects for the uncorrected data are 1557 ± 377 ms, for the corrected data 1283 ± 33 ms, for the breathhold data 1240 ± 57 ms and for MOLLI 1233 ± 26. Motion correction resulted in T1 values being more uniform regarding the T1 variation in the segments as for the uncorrected T1 maps. In Fig. 6c the differences of the T1 values are compared against the reference breathhold method.

**Fig. 7:**
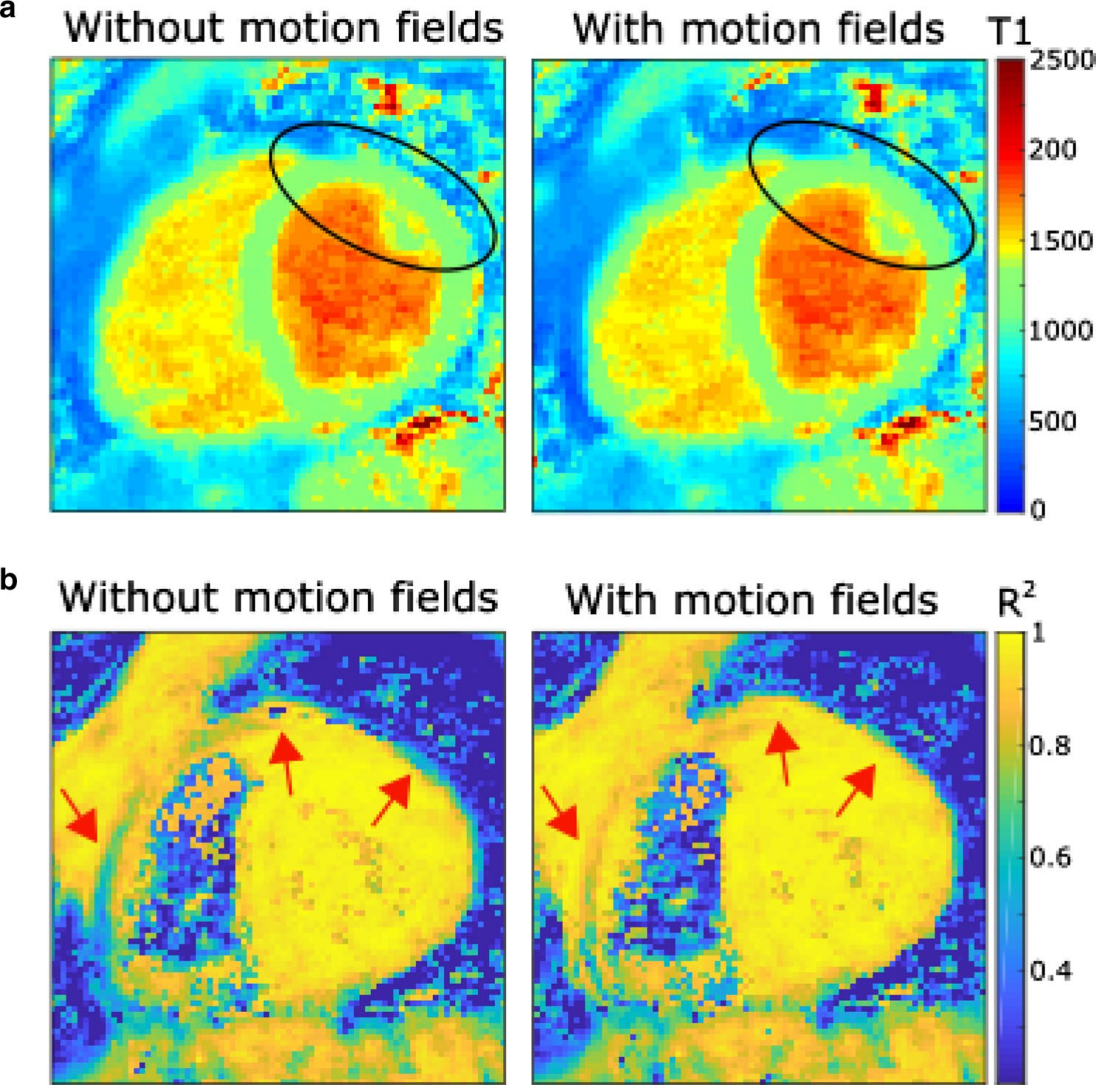
**a** T1 maps of a subject without motion fields and with motion fields. **b** *R*^2^ maps of the same subject with arrows pointing at regions, where the motion fields result in improvements of the fit.

Figure 7 shows the T1 maps of a subject and the corresponding *R*^2^ of the model fit with and without retrospective non-rigid motion correction. Although the differences in T1 are very small between these two reconstructions, the non-rigid motion correction leads to an improvement in *R*^2^ especially along the edges of the myocardium suggesting a better alignment between the images used for the T1 fit.

Cine images with 28 ± 3 heart phases were reconstructed from the same k-space data as the T1 maps. The supplementary video shows cine images with and without motion correction of three subjects together with breathhold data and the reference standard GRE cine data. With motion correction, respiratory artifacts were strongly reduced and the visibility of the myocardium was improved. This enabled accurate determination of static periods in cardiac cycle.

**Fig. 8:**
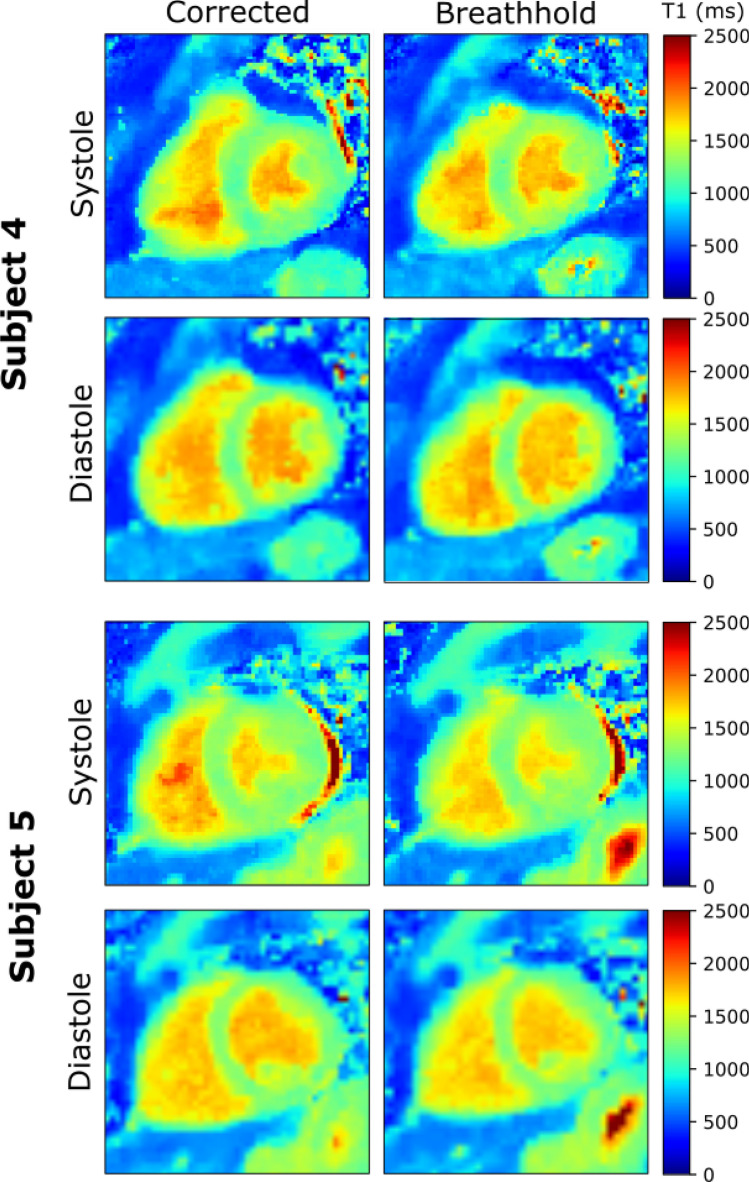
Native T1 maps of systolic and diastolic heart phases of two subjects with motion correction (corrected) and for comparison of breathhold data acquired during a separate scan

Furthermore, the T1 maps of two subjects estimated for systole and diastole with motion correction and during a breathhold are shown in Fig. 8. Cine images were used as a scout in order to identify peak systole and optimize the reconstruction window to minimize cardiac motion artifacts. 30 and 23 radial lines per image, corresponding to a reconstruction window of 141 and 108 ms, were used for subject 4 and 5, respectively. The same window duration for systolic and diastolic static periods were used in this figure for better comparability.

**Fig. 9:**
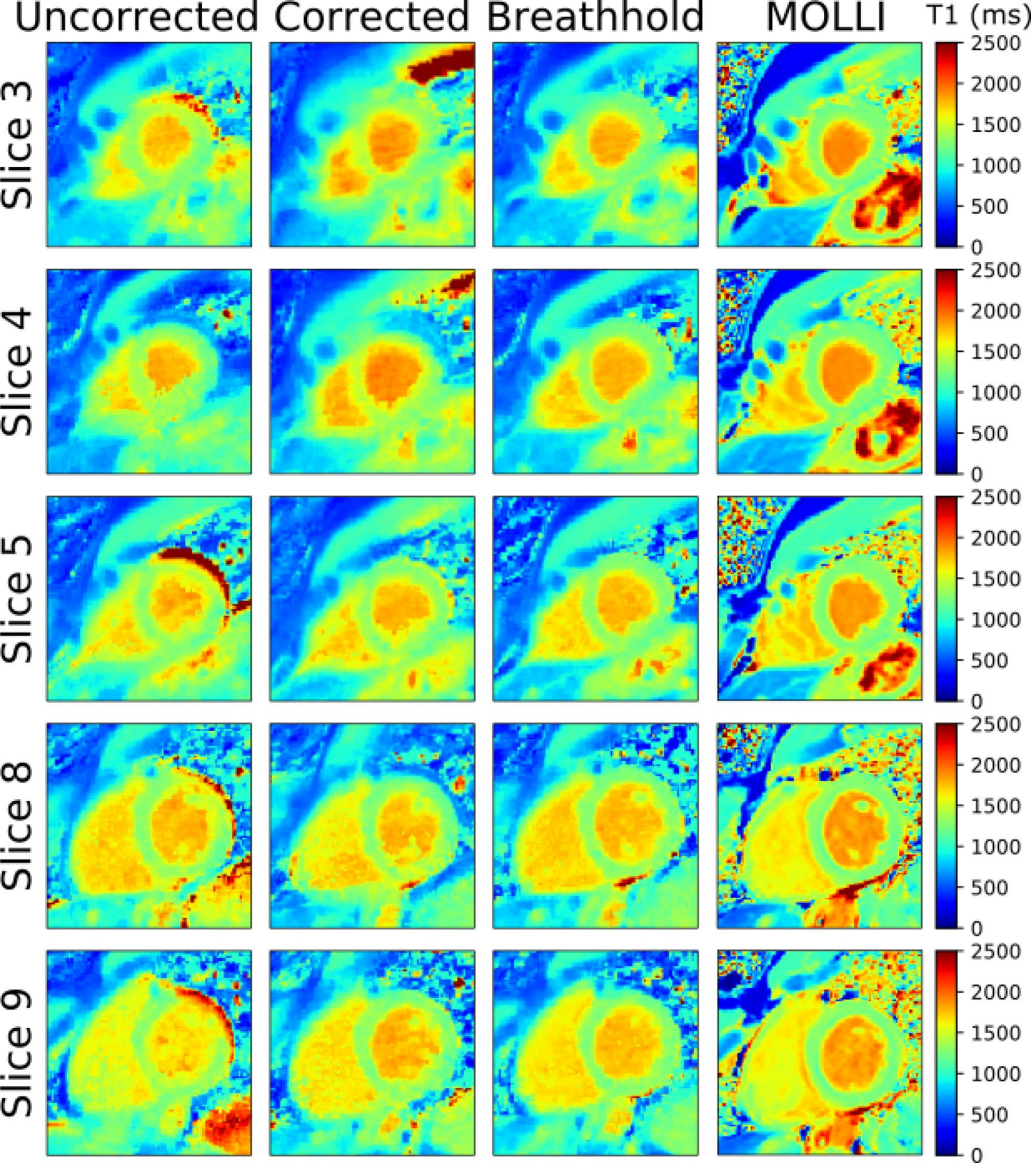
Short axis T1 maps in one healthy subject. 10 slices (5 displayed) were recorded, covering the complete left ventricle. The same calibration data was used for all corrected images. For comparison, resulting maps of a breathhold scan and MOLLI are displayed. Motion correction led to a reduction of respiratory artifacts. Each method was acquired in a separate scan resulting in small differences in the visualized anatomy

Figure 9 shows five out of 10 T1 maps of one subject covering the left ventricle in short axis orientation acquired during free-breathing without correction and with correction. The motion correction was based on the same calibration scan for all slices. Again, for comparison, data were also acquired during a single breathhold and using MOLLI as a visual reference.

**Fig. 10:**
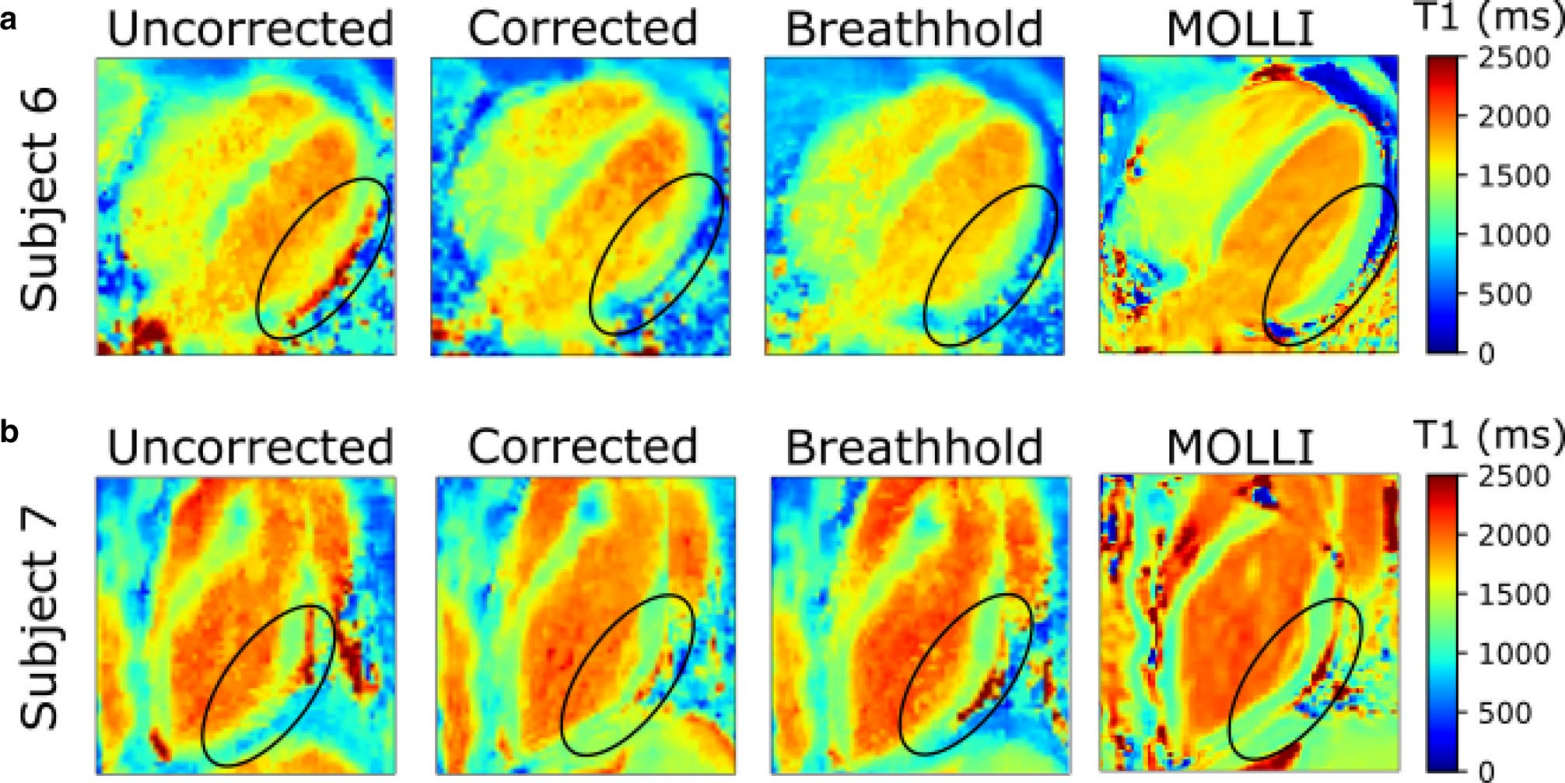
**a** Mid-diastole in 4CHV and **b** LA for two subjects acquired during free-breathing with and without correction. For comparison, breathhold data is also shown together with MOLLI data. The black circles highlight the lateral wall of the left ventricle.

In Fig. 10, T1 maps of two different subjects in 4CHV and LA are shown with and without motion correction under free-breathing conditions. For comparison, a breathhold scan and a MOLLI T1 map are also displayed. The data were recorded in separate scans, thus there might be slight differences in the T1 maps. The T1 values in the lateral wall of the left ventricle, as highlighted with black circles appear more uniform with motion correction than without motion correction.

## Discussion

Phantom and in vivo measurements demonstrated the improvement in both image quality and T1 quantification using the proposed motion correction approach compared to motion uncorrected imaging.

In a phantom study, T1 values obtained with our approach showed accurate T1 quantification over a wide range of T1 times. Changes of the phantom over time due to motion led to inaccurate T1 estimation, especially in areas where there are large differences between neighboring T1 values. The material, surrounding the nine tubes, had a low T1 time of 713 ± 23 ms. Therefore, the error of T1 quantification was highest for tubes with a high T1 value.

The cine images reconstructed from the continuous data acquisition showed poorer image quality compared to a conventional cine scan. Nevertheless, they provided cardiac motion information which allowed for the retrospective optimization of the data used for T1 mapping.

One advantage of the sequence used here compared to an ECG-triggered acquisition is that data from all cardiac phases can be recorded in a very short time. Cardiac multi-phase T1 maps can be reconstructed by optimizing the cardiac phase retrospectively via cardiac gating. In this paper T1 maps of mid-diastole and mid-systole were reconstructed. Also, it was previously reported that MOLLI underestimates T1 values, due to heartrate dependency and T2 [46, 51–54]. Therefore, more accurate T1 sequences should be used.

The highest errors in T1 quantification were found in the anterior and anterolateral segments which border to the lung and showed very large signal differences. Significant differences were found between the uncorrected scan and the breathhold scan for all segments except the inferoseptal segment. Although motion artifacts are not immediately visible in the T1 maps, respiratory motion still impacted T1 quantification. The motion-corrected T1 maps showed very similar T1 values to the breathhold scan, except for one segment. However, in this anteroseptal segment T1 values were lower in the breathhold scan compared to the other segments.

The calibration scan was performed in a midventricular ROI. As a result, the motion of the midventricular area was estimated most accurately. Still, high image quality could be seen for all short axis images in the full stack of slices of a subject, covering 8 cm along the long axis of the heart. For the depicted LA and 4CHV T1 maps, artifacts in the lateral wall were decreased with the motion correction. For these orientations, the motion field estimation parameters could be adjusted and tested on more subjects in the future.

One of the limitations of this study is that the rigid motion correction was only performed for HF and AP direction (i.e., the two prominent directions of motion [55]). Only sagittal images were acquired for calibration to keep the processing time for the derivation of motion models short. Additional correction of RL motion could further improve image quality.

Also, we used the same motion model for inspiration and expiration and did not explore the effect of hysteresis. Nevertheless, previous studies have shown that these effects are small [31]. Still, more advanced models, such as affine motion models or taking hysteresis effects between inspiration and expiration into account, could improve the prediction of respiratory motion and image quality [56–58].

Depending on the subject and the selected slice orientation, the motion amplitude varies. The determined motion model and correction parameters were only valid for the chosen heart region but were applied globally to all image data. The surrounding static tissue (e.g., back and spine) or tissue that moves differently than the heart (e.g., liver) are wrongly corrected, which can result in artifacts. However, for radial trajectories, these artifacts mainly lead to streaking artifacts which did not impair the T1 estimation of the heart in this study.

The residual motion fields after PT-based rigid motion correction in the cardiac region were on average small (1.1 ± 0.4 mm) which indicated that the PT-based motion correction was already very accurate. The two main sources of residual motion are due to inaccuracies in our linear motion model leading to incomplete correction of respiratory motion and the missing right-left motion component in our motion model. The additional motion correction with the motion fields did further minimize residual motion and improve T1 fitting. Correction of in-plane respiratory motion is applied retrospectively, and motion fields were estimated from the same data that was used for T1 mapping. Hence, this step did not require any additional scan time. Studies in patients who may have more complicated cardiac motion due to disease are still needed to assess the applicability of this approach to routine clinical practice.

In this paper, a PT based motion correction approach with additional non-rigid motion field correction for free-breathing T1 mapping with continuous radial trajectory was presented. To the authors knowledge, so far, no method has been presented that performs prospective through-plane motion correction for T1 mapping. 

The introduced tools will enable free-breathing T1 mapping with respiratory and cardiac motion correction as well as free-breathing 3D data acquisition in the future. The PT could also be used for other MR imaging strategies for the heart that are additionally limited by breathhold duration (e.g., perfusion or MRF).

## Conclusion

In our study, we demonstrated that the PT is suitable for prospective respiratory motion correction for free-breathing myocardial T1 mapping using a radial acquisition trajectory. Our method was tested on a motion phantom and showed an improvement in the T1 estimation accuracy compared to uncorrected data. Also, in the in vivo measurements during free-breathing, our method was able to provide more accurate T1 values in the myocardium compared to the uncorrected scans. Further studies are needed to confirm our method in clinical practice.

## Supplementary Information

Below is the link to the electronic supplementary material.

Supplementary Video Cine images obtained from radial T1 mapping sequence with and without motion correction compared to the breath hold acquisition and a standard GRE cine for 3 subjects (AVI 356 KB)
